# Limb Salvage in Myxoid Liposarcoma With Tumor Prosthetic Reconstruction: A Case Report and Literature Review

**DOI:** 10.7759/cureus.111789

**Published:** 2026-06-30

**Authors:** Alberto Olan-Rovirosa, Omar Fernando Rodriguez-Rodriguez, Mario J.P. Gallegos-Alvarado, Othoniel Cruz-Jimenez, Eduardo Wong-Castrejon

**Affiliations:** 1 Orthopedics and Traumatology Department, Instituto de Seguridad y Servicios Sociales de los Trabajadores del Estado (ISSSTE) Hospital Regional Monterrey, Monterrey, MEX

**Keywords:** bone tumors, limb salvage, myxoid liposarcoma, quality of life (qol), tumoral prosthesis

## Abstract

Myxoid liposarcoma (MLPS) is the second most common histological subtype of liposarcoma and typically affects the deep soft tissues of the lower extremities. Although local recurrence rates are relatively low, delayed recurrence and metastatic disease may occur years after initial treatment. Pathologic fractures associated with extensive femoral involvement are uncommon and represent a major reconstructive challenge. We present the case of a 62-year-old female with a history of right proximal femoral MLPS treated with radiotherapy 12 years earlier who presented with progressive right hip pain and inability to bear weight following minor trauma. Imaging studies demonstrated a basicervical pathologic fracture associated with extensive osteolytic involvement of the proximal and diaphyseal femur. Due to severe functional deterioration and extensive bone destruction, the patient underwent en bloc femoral resection, followed by reconstruction with a modular total femoral tumor prosthesis as part of a limb salvage procedure. Histopathological evaluation using hematoxylin and eosin staining demonstrated recurrent undifferentiated MLPS with medullary cavity infiltration. Immunohistochemical and cytogenetic studies were unavailable because of institutional resource limitations. At six months of follow-up, the patient remained ambulatory with cane assistance, demonstrated satisfactory pain control, stable implant positioning, and no evidence of mechanical failure. This case highlights the feasibility of limb salvage surgery with total femoral reconstruction in selected patients with recurrent MLPS presenting with pathologic fracture and extensive femoral involvement. Multidisciplinary management and individualized surgical planning remain essential to optimize oncologic and functional outcomes.

## Introduction

Myxoid liposarcoma (MLPS) is a malignant adipocytic neoplasm representing approximately 30% of all liposarcomas and nearly 10% of soft tissue sarcomas (STSs) [1]. It is considered the second most common histological subtype of liposarcoma and typically arises in the deep soft tissues of the lower extremities, particularly in the thigh. Magnetic resonance imaging (MRI) remains the imaging modality of choice for local tumor characterization and surgical planning [2]. Although MLPS generally demonstrates favorable sensitivity to radiotherapy and relatively low local recurrence rates, metastatic disease and delayed recurrence may occur several years after initial treatment [3]. Positive surgical margins have been associated with increased local recurrence rates, while negative margins significantly improve local disease control. Consequently, multimodal treatment strategies involving surgical resection, radiotherapy, and systemic therapy are frequently required [4].

Historically, amputation represented the standard treatment for locally advanced extremity STSs. However, advances in oncologic surgery, imaging, chemotherapy, radiotherapy, and endoprosthetic reconstruction have progressively shifted management toward limb salvage procedures, allowing preservation of function while maintaining acceptable oncologic outcomes [5,6].

We present a rare case of recurrent MLPS presenting as a pathologic proximal femoral fracture managed with en bloc resection and total femoral tumor prosthetic reconstruction.

## Case presentation

A 62-year-old female patient from Monterrey, Nuevo León, Mexico, presented to our institution with progressive right hip pain of five months' duration following a fall from standing height. The patient had a history of MLPS involving the right proximal femur and hip region, diagnosed 12 years earlier (2012), which had been treated with radiotherapy for three years with oncology follow-up. No additional significant medical or surgical history was identified on direct questioning.

The patient reported progressive pain intensity until functional limitation became disabling. Upon admission to the orthopedics department, she described severe right inguinal pain rated 8/10 on the visual analog scale (VAS) [7], partially relieved with intravenous opioid analgesics and exacerbated by passive mobilization of the right lower extremity. The patient was unable to bear weight.

Physical examination demonstrated a 1-cm shortening of the right lower extremity, edema over the proximal femur, tenderness on palpation of the right groin, and severely limited active and passive hip range of motion secondary to pain. Neurological examination of the right lower extremity demonstrated muscle strength graded as 3/5 at L3-L4 and 4/5 at L5-S1 according to the Daniels scale [8], with preserved sensation in L2-S1 dermatomes. Distal perfusion was preserved with a capillary refill <2 seconds and no evidence of vascular compromise. Examination of the contralateral lower extremity was unremarkable.

Diagnostic assessment

Initial evaluation included anteroposterior radiographs of the pelvis and right hip, as well as staging thoracoabdominopelvic computed tomography (CT) with extension to the lower extremities to evaluate for distant disease.

Radiographs demonstrated a complete basicervical fracture of the right femur associated with varus collapse of the femoral neck and extensive osteolytic lesions involving the right iliac bone and proximal femur, suggestive of a pathologic fracture (Figures 1-2). These lesions demonstrated heterogeneous intraosseous involvement with septated lytic changes disrupting the normal trabecular architecture, extending from the proximal femur to the distal metaphyseal-diaphyseal junction, without evidence of periosteal reaction or apparent soft tissue extension.

**Figure 1:**
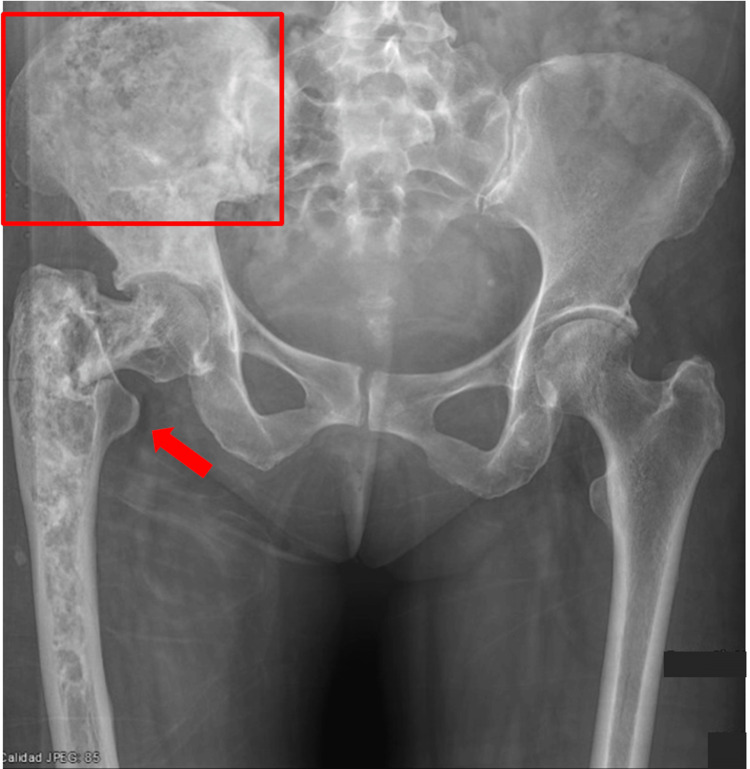
Admission anteroposterior radiograph of the pelvis. A discontinuity in the cortical bone is observed at the base of the right femoral neck, causing displacement of the proximal fragment in abduction and elevation, with impaction of the distal fragment. An infiltrative pattern with a moth-eaten appearance is seen affecting the head, neck, and diaphysis of the femur, with thinning of the cortex of the right femur, predominantly in the proximal region (arrows). In addition, adjacent infiltration is observed in the left iliac bone and acetabulum (red square).

**Figure 2:**
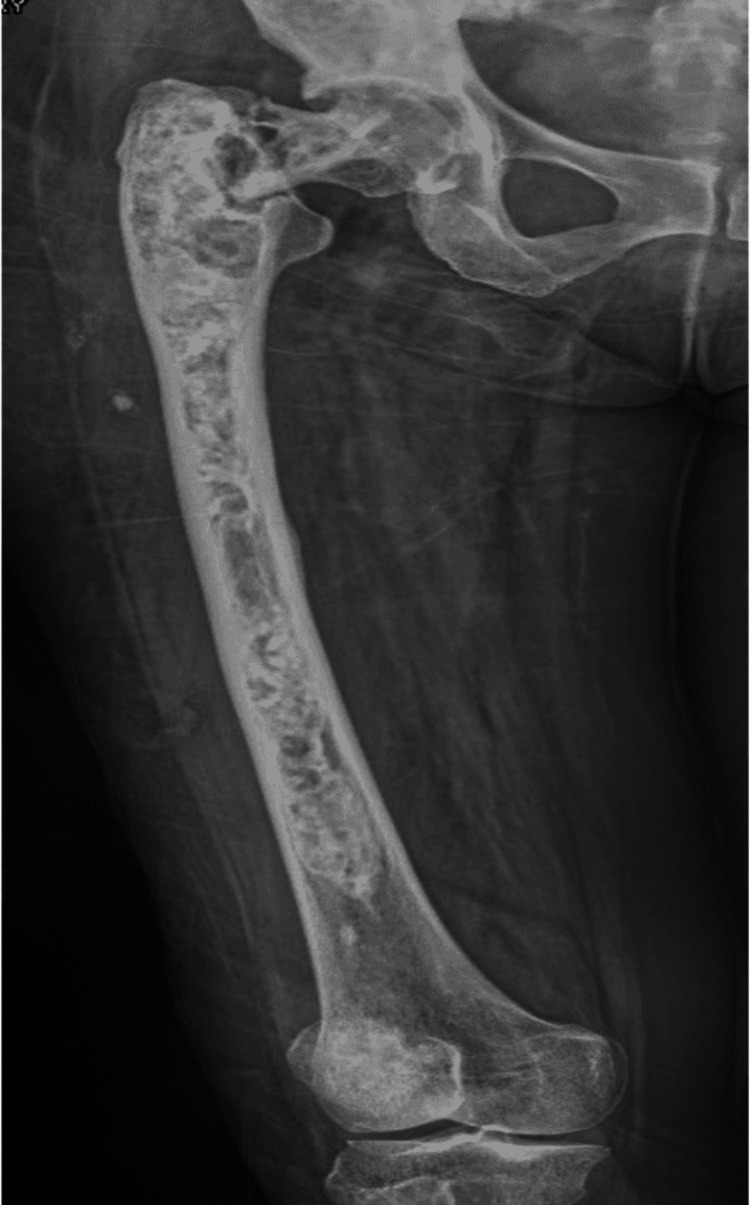
Admission anteroposterior radiograph of the right femur. A moth-eaten pattern is observed involving the medullary canal in the proximal two-thirds of the femur, with cortical thinning in the proximal region and loss of cortical continuity at the base of the femoral neck.

CT confirmed the presence of a basicervical proximal femoral fracture associated with heterogeneous intramedullary lesions involving the proximal and diaphyseal femur. The lesions demonstrated multiple septations and variable intramedullary densities with destruction of normal trabecular bone architecture. No extraosseous disease or distant metastatic lesions were identified on staging imaging (Figure 3).

**Figure 3:**
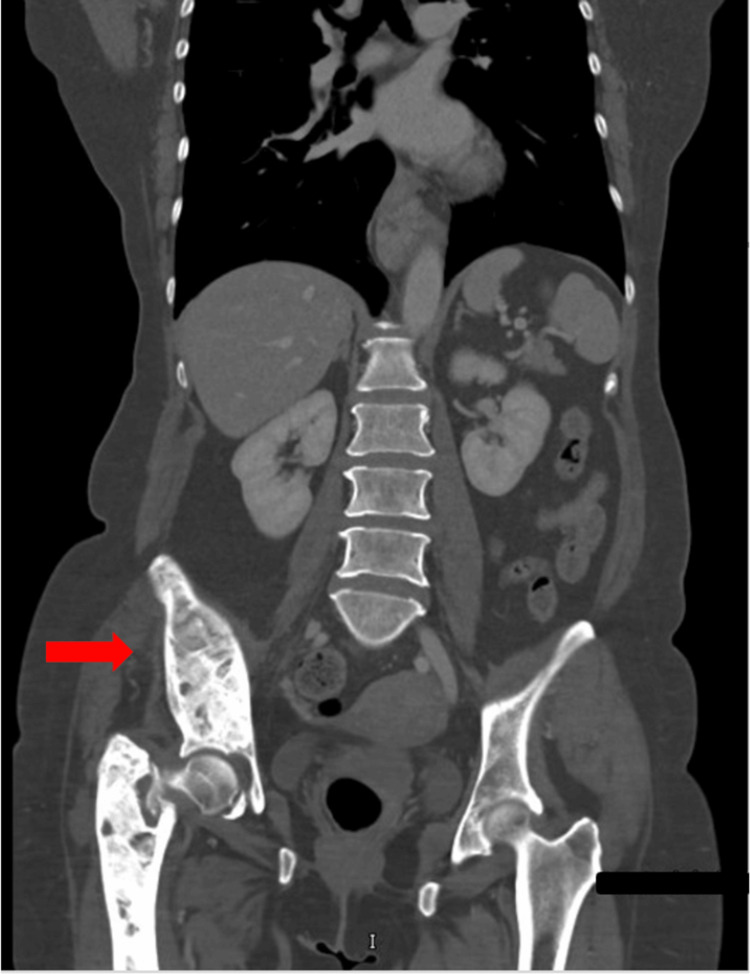
Admission thoracoabdominopelvic computed tomography, bone window, coronal view. Loss of bone continuity is observed at the base of the femoral neck, associated with bone infiltration affecting the right iliac bone (arrow) and the right femur. No infiltration of adjacent organs is observed.

Given the patient's severe functional deterioration, extensive osseous involvement, and impending mechanical instability, definitive surgical management was prioritized. Preoperative MRI and biopsy were limited by institutional resource availability and the urgent need for definitive stabilization and oncologic resection.

Therapeutic intervention

The patient was admitted to the orthopedics service for pain control and preoperative optimization. An internal medicine evaluation was performed to improve her general medical condition prior to surgery.

Five days after admission, surgical treatment was performed using a direct lateral approach to the right hip. Layer-by-layer dissection was carried out until exposure of the femoral diaphysis was achieved. Preservation of the acetabular capsule was attempted whenever oncologically feasible. The abductor mechanism and psoas tendon were carefully identified and isolated.

Intraoperative fluoroscopic evaluation demonstrated extensive tumoral involvement extending to the supracondylar region of the femur. En bloc resection of the proximal and diaphyseal femur was subsequently performed until macroscopically viable margins were obtained, confirmed by an intraoperative biopsy.

Following complete femoral resection, reconstruction was achieved using a modular total femoral tumor prosthesis (Figures 4-5). Cerclage fixation and structural graft augmentation were utilized to reinforce proximal fixation and improve implant stability. Reattachment of the psoas tendon and abductor mechanism was performed through the fenestrated components of the prosthesis to optimize postoperative function and soft tissue balance.

**Figure 4:**
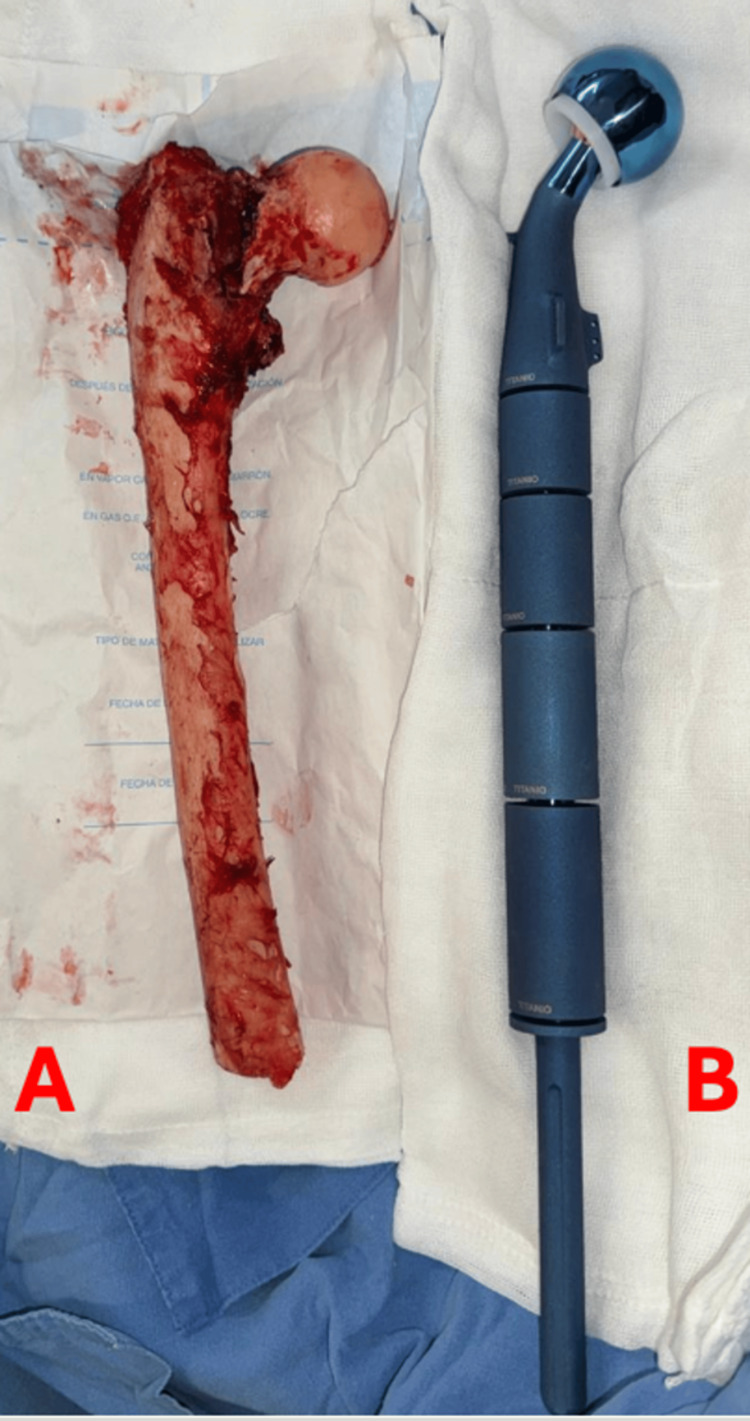
Intraoperative en bloc resection. The en bloc resection of the affected right femur is shown (A), as well as the tumoral bipolar proximal femoral prosthesis used in the surgery (B), in which the prosthetic modules are visible.

**Figure 5:**
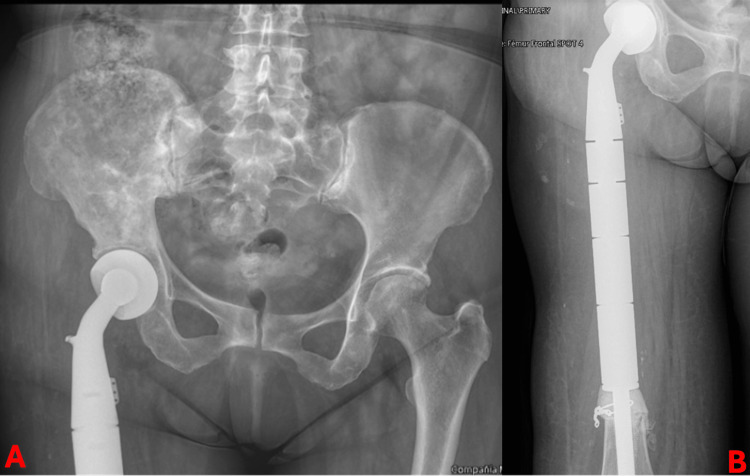
Immediate postoperative radiographs following total femoral reconstruction. (A) Anteroposterior pelvis radiograph demonstrating adequate positioning and alignment of the proximal component of the modular total femoral tumor prosthesis, with preservation of hip joint congruency. (B) Anteroposterior radiograph of the right femur demonstrating satisfactory alignment of the femoral reconstruction, stable implant fixation, and absence of immediate postoperative mechanical complications.

One unit of packed red blood cells was transfused intraoperatively. The surgical specimen was sent for definitive histopathological analysis. 

Histopathology

Macroscopic examination of the en bloc surgical specimen demonstrated a 30 × 5 × 4.8 cm resection specimen, including the femoral head and extending to the distal metaphyseal region of the femur. Sectioning revealed a heterogeneous intramedullary tumor measuring approximately 27 × 2.5 cm with irregular borders and semisolid myxoid characteristics occupying the medullary canal of the proximal and diaphyseal femur.

Microscopic evaluation demonstrated neoplastic infiltration of the medullary cavity composed of variable-sized adipocytes embedded within a myxoid stroma containing small neoplastic cells with hyperchromatic nuclei, findings compatible with recurrent MLPS (Figure 6). Distal surgical margins were initially reported as microscopically positive.

**Figure 6:**
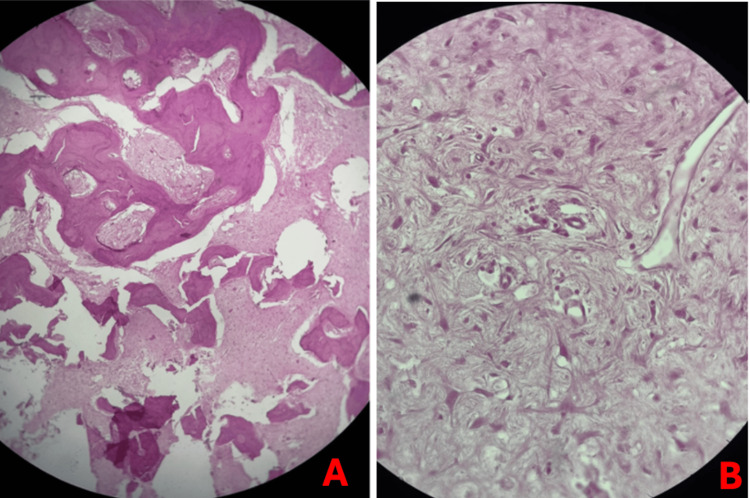
Histopathological section stained with hematoxylin and eosin. (A) Low-power microscopic view demonstrating trabecular bone infiltration and replacement of the normal medullary architecture by neoplastic tissue. (B) High-power view showing undifferentiated neoplastic cells embedded within a myxoid stroma, composed of spindle-shaped and pleomorphic cells with hyperchromatic nuclei, consistent with recurrent myxoid liposarcoma.

Histological staining was performed with hematoxylin and eosin (H&E). The lesion demonstrated undifferentiated histological features. Immunohistochemical analysis and cytogenetic studies for translocation assessment were not performed due to institutional resource limitations.

Follow-up

Postoperative hospital care consisted of 72-hour monitoring focused on hemodynamic stability, wound surveillance, and pain control. The patient progressed favorably without immediate postoperative complications.

Partial weight-bearing of the right lower extremity was initiated, and a knee extension brace was indicated during the first postoperative month. At the one-month follow-up visit, the patient was ambulating with the assistance of a cane and reported adequate pain tolerance. Surgical wound healing progressed uneventfully, and follow-up radiographs demonstrated appropriate implant positioning without evidence of loosening or mechanical failure (Figure 7).

**Figure 7:**
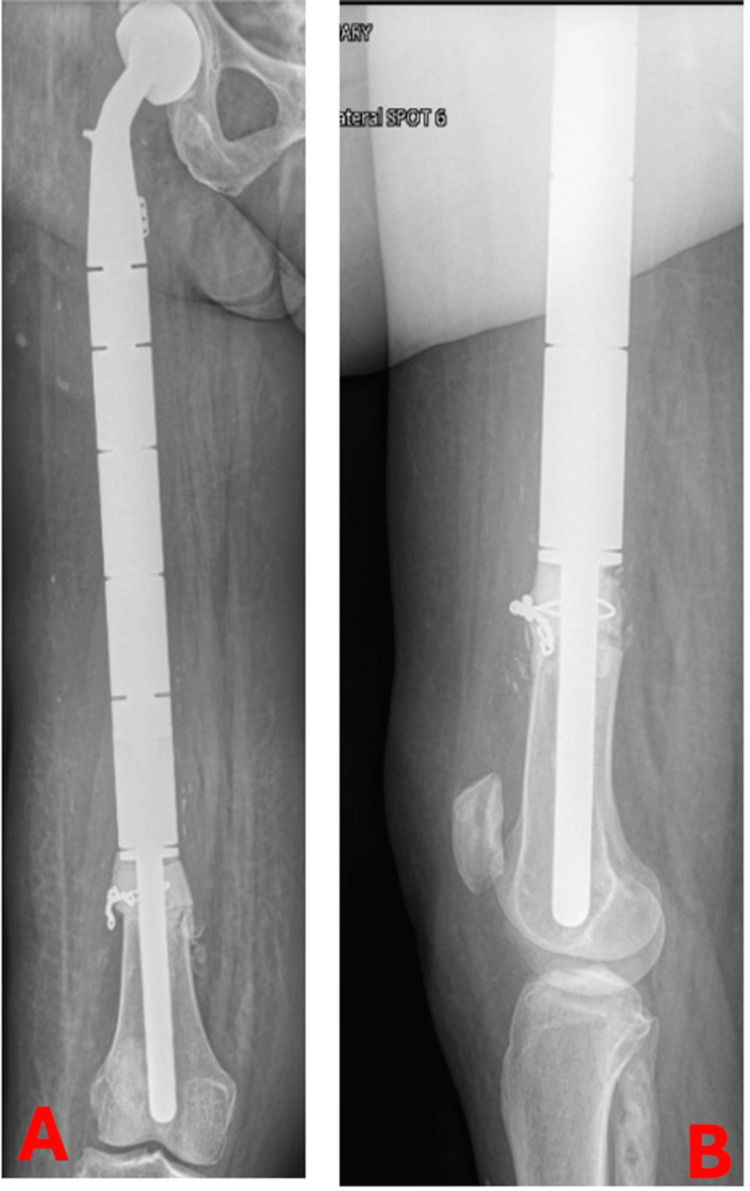
One-month follow-up radiographs following total femoral reconstruction. (A) Anteroposterior radiograph of the right femur demonstrating maintained alignment and stable fixation of the modular total femoral tumor prosthesis without evidence of implant loosening or periprosthetic complications. (B) Lateral radiograph demonstrating adequate prosthetic positioning, preserved distal fixation, and satisfactory postoperative alignment at the knee joint.

At six months of follow-up, the patient remained ambulatory with cane assistance and demonstrated adequate tolerance to prolonged walking without significant pain. Gait was stable, without evident limping or compensatory mechanisms (Figure 8).

**Figure 8:**
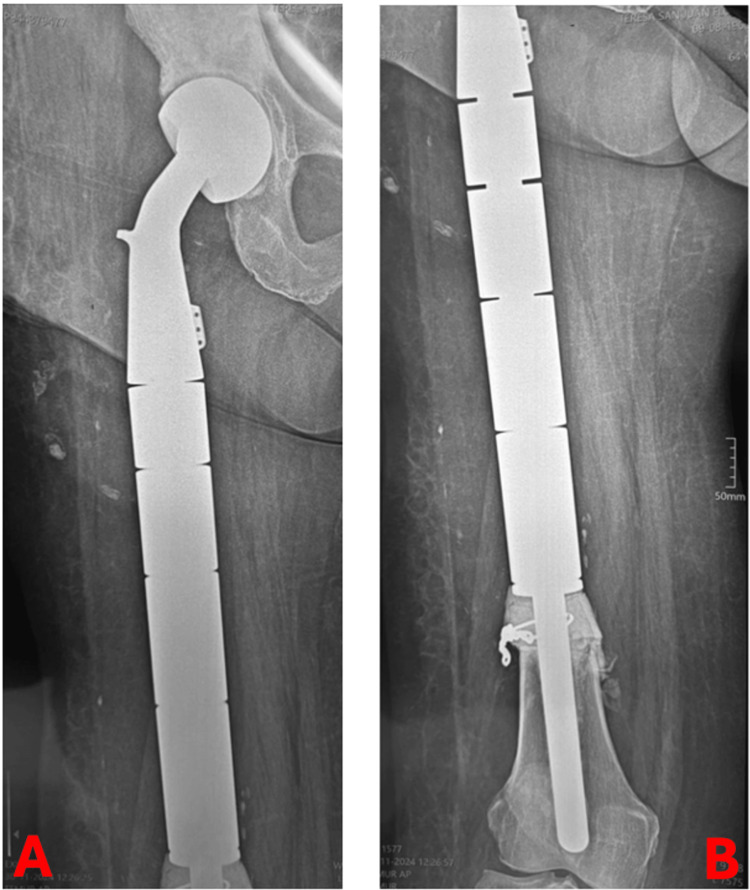
Six-month follow-up radiographs following total femoral reconstruction. (A) Anteroposterior radiograph of the proximal femoral reconstruction demonstrating maintained prosthetic alignment and stable acetabular articulation without evidence of loosening or migration. (B) Anteroposterior radiograph of the distal femoral component demonstrating preserved implant stability, maintained alignment, and absence of periprosthetic fracture or mechanical failure at final follow-up.

Inspection of the surgical site demonstrated a well-healed incision without signs of infection, erythema, drainage, or wound complications. Neurological and distal vascular examinations remained within normal limits.

Range of motion assessment of the right knee demonstrated preserved full extension (180°) with flexion limited to 70°. Passive and active movements were concordant and painless, with no evidence of instability on ligamentous examination. The patient was referred to an intensive and comprehensive physical rehabilitation program consisting of five sessions per week, which resulted in an improve of range of motion being achieved after three months.

Despite the favorable oncologic and functional evolution, postoperative knee stiffness emphasized the importance of early and aggressive rehabilitation protocols following extensive limb salvage reconstruction.

## Discussion

MLPS is one of the most common histological subtypes of liposarcoma; however, osseous involvement associated with pathologic fracture remains uncommon [9-12]. Furthermore, delayed recurrence and metastatic presentation may occur several years after the initial diagnosis, as observed in the present case, in which recurrence developed approximately 12 years after primary treatment [13,14].

The management of recurrent MLPS involving extensive femoral destruction presents a significant surgical challenge. Historically, amputation was considered the standard treatment for advanced extremity soft tissue sarcomas to ensure adequate local control [15]. Nevertheless, advances in oncologic reconstruction techniques have progressively expanded the indications for limb salvage procedures.

Several authors have reported successful management of MLPS using wide surgical excision combined with adjuvant radiotherapy and chemotherapy. Xu et al. described successful en bloc resection and radiotherapy in a patient with MLPS involving the popliteal fossa, while other reports have demonstrated favorable oncologic outcomes following aggressive surgical management in anatomically complex locations [16-18] (Table 1).

**Table 1:** Cases of myxoid liposarcoma and management strategies. MLPS: myxoid liposarcoma

Author/year	Age	Affected zone	Diagnosis	Tumor size	Management/treatment
Wang et al. [13]	40 years	Body of the pancreas	MLPS	14 × 9 × 7 cm	Middle pancreatectomy and pancreaticojejunostomy + radiotherapy
Ciongariu et al. [14]	32 years	Left thigh	Dedifferentiated liposarcoma arising from MLPS	11 × 7 × 2 cm	Wide local excision with clear resection margins and lymphadenectomy
Rico Gala et al. [15]	57 years	Right parauterine mass	High-grade MLPS	6.6 × 7.1 × 7 cm	Lesion excision
Xu et al. [16]	35 years	Right popliteal fossa	MLPS	20 × 17 × 10 cm	En bloc resection + radiotherapy
Finlay et al. [17]	51 years	Left thigh	High-grade MLPS	26 × 23 × 36 cm	En bloc resection + delayed plastic reconstruction + chemotherapy + radiotherapy
Kim et al. [18]	60 years	Small bowel mesentery	Metastatic MLPS	25 × 20 cm	Wide local excision and small bowel resection + doxorubicin-based chemotherapy

In the present case, the extensive involvement of the proximal and diaphyseal femur associated with a pathologic fracture required radical oncologic resection, followed by total femoral reconstruction using a modular tumor prosthesis. Despite the complexity of the procedure, limb salvage allowed preservation of ambulation and acceptable postoperative function.

An additional challenge during management was the limited availability of specialized oncologic implants within our institution. Although our center functions as a tertiary referral hospital capable of managing complex orthopedic oncology cases, the acquisition of the required prosthesis required administrative authorization through federal institutional channels due to the high cost and specialized nature of the implant.

The absence of intraoperative frozen section analysis represented another important limitation. Consequently, final margin assessment was only available postoperatively, initially demonstrating positive distal microscopic margins. Nevertheless, the patient subsequently underwent adjuvant systemic therapy, and a repeat percutaneous biopsy performed two months later demonstrated negative residual disease.

Current evidence suggests that multidisciplinary management involving orthopedic oncology, pathology, radiology, medical oncology, and rehabilitation is essential to optimize both oncologic and functional outcomes in patients with MLPS. Prognostic factors, including tumor size, histologic grade, metastatic disease, and surgical margins, remain critical determinants of long-term survival.

Compared with segmental resection or amputation, total femoral reconstruction was considered the most appropriate option because of the extensive femoral involvement extending from the proximal femur to the distal metaphyseal-diaphyseal junction. This approach allowed complete tumor resection while preserving the limb, maintaining ambulatory function, and avoiding the functional limitations associated with amputation. In carefully selected patients, limb salvage can provide satisfactory functional outcomes without compromising oncologic principles.

## Conclusions

This case highlights the feasibility of limb salvage surgery with total femoral tumor prosthetic reconstruction in selected patients with recurrent MLPS presenting with pathologic fracture and extensive femoral involvement.

Despite significant oncologic and reconstructive challenges, multidisciplinary management and individualized surgical planning allowed preservation of limb function and satisfactory short-term clinical outcomes. Total femoral reconstruction may represent a viable alternative to amputation in carefully selected patients with advanced extremity sarcomas.
